# Encapsulation of *Lactobacillus reuteri* in Chia–Alginate Hydrogels for Whey-Based Functional Powders

**DOI:** 10.3390/gels11080613

**Published:** 2025-08-04

**Authors:** Alma Yadira Cid-Córdoba, Georgina Calderón-Domínguez, María de Jesús Perea-Flores, Alberto Peña-Barrientos, Fátima Sarahi Serrano-Villa, Rigoberto Barrios-Francisco, Marcela González-Vázquez, Rentería-Ortega Minerva

**Affiliations:** 1 Tecnológico Nacional de México/TES de San Felipe del Progreso, San Felipe del Progreso 50640, Mexico; yadiscica19@gmail.com (A.Y.C.-C.); rigoberto.bf@sfelipeprogreso.tecnm.mx (R.B.-F.); 2 Departamento de Ingeniería Bioquímica, Escuela Nacional de Ciencias Biológicas, Instituto Politécnico Nacional, Ciudad de México 07738, Mexico; gcalderon@ipn.mx (G.C.-D.); fserranov2001@alumno.ipn.mx (F.S.S.-V.); 3 Centro de Nanociencias y Micro y Nanotecnologías, Instituto Politécnico Nacional, Ciudad de México 07738, Mexico; mpereaf@ipn.mx (M.d.J.P.-F.); apenab@ipn.mx (A.P.-B.); 4 Instituto de Farmacología, Universidad de la Cañada, Carretera Teotitlan—San Antonio Nanahuatipan Km 1.7 s/n, Paraje Titlacuatitla, Teotitlan de Flores Magón, Oaxaca 68540, Mexico; marcelaglezvaz89@hotmail.com

**Keywords:** encapsulation, *Lactobacillus reuteri DSM 17938*, electrospraying, whey

## Abstract

This study aimed to develop a functional powder using whey and milk matrices, leveraging the protective capacity of chia–alginate hydrogels and the advantages of electrohydrodynamic spraying (EHDA), a non-thermal technique suitable for encapsulating probiotic cells under stress conditions commonly encountered in food processing. A hydrogel matrix composed of chia seed mucilage and sodium alginate was used to form a biopolymeric network that protected probiotic cells during processing. The encapsulation efficiency reached 99.0 ± 0.01%, and bacterial viability remained above 9.9 log_10_ CFU/mL after lyophilization, demonstrating the excellent protective capacity of the hydrogel matrix. Microstructural analysis using confocal laser scanning microscopy (CLSM) revealed well-retained cell morphology and homogeneous distribution within the hydrogel matrix while, in contrast, scanning electron microscopy (SEM) showed spherical, porous microcapsules with distinct surface characteristics influenced by the encapsulation method. Encapsulates were incorporated into beverages flavored with red fruits and pear and subsequently freeze-dried. The resulting powders were analyzed for moisture, protein, lipids, carbohydrates, fiber, and color determinations. The results were statistically analyzed using ANOVA and response surface methodology, highlighting the impact of ingredient ratios on nutritional composition. Raman spectroscopy identified molecular features associated with casein, lactose, pectins, anthocyanins, and other functional compounds, confirming the contribution of both matrix and encapsulants maintaining the structural characteristics of the product. The presence of antioxidant bands supported the functional potential of the powder formulations. Chia–alginate hydrogels effectively encapsulated *L. reuteri*, maintaining cell viability and enabling their incorporation into freeze-dried beverage powders. This approach offers a promising strategy for the development of next-generation functional food gels with enhanced probiotic stability, nutritional properties, and potential application in health-promoting dairy systems.

## 1. Introduction

In recent years, the primary purpose of foods has evolved from fulfilling their basic nutritional role to providing health and well-being benefits, thereby driving the growth of the functional food and probiotic industry [1]. In this sense, the International Life Sciences Institute (ILSI) defines a functional food as “A food that contains a component, nutrient, or non-nutrient, with an effect on one or more functions of the body, with an added effect beyond its nutritional value” [2]. Within this context of development, the incorporation of probiotic strains into food matrices has gained significant importance as they must withstand processing conditions to ensure a sufficient quantity of viable cells (≥10^6^ CFU/g) at the time of consumption, thus guaranteeing their functional effect. Functional foods often incorporate probiotics, as the inclusion of beneficial live microorganisms is one of the most effective ways to enhance the beneficial properties of food products.

Considering the above, some probiotic microorganisms, such as *Lactobacillus reuteri*, contribute to the balance of the intestinal microbiota, immune modulation, and the prevention of gastrointestinal disorders, thus reinforcing the functional attributes of the food products that deliver them. Accordingly, probiotics have become a central element in the formulation of dairy products, beverages, and functional supplements, representing a rapidly growing sector of the food industry. However, their integration into food matrices requires strategies that guarantee their viability and stability during processing and storage. However, achieving high levels of viability represents a challenge for the food industry due to the sensitivity of probiotics to adverse factors such as temperature and oxygen during processing [3]. Therefore, the use of encapsulation techniques that protect probiotic cells and promote their stability and functionality has become essential.

Encapsulation is a technique that has been established as a strategy to improve the survival of probiotic microorganisms against adverse conditions in food and during their passage through the gastrointestinal tract, enhancing cell viability by retaining the bacteria within polymeric matrices that act as semipermeable physical barriers, regulating the interaction between the probiotic and the environment. Currently, extrusion, ionic gelation, and spray drying are the most commonly used methods for probiotic encapsulation. However, these methods present several limitations due to poor control of particle size, low uniformity in the microcapsules, and exposure to high temperatures during the process.

Despite the growing demand for functional foods enriched with probiotics, the industry continues to face major challenges related to the survival of these microorganisms during food processing, storage, and gastrointestinal transit. Conventional encapsulation methods often expose probiotics to high temperatures or produce irregular microcapsules with poor protective capacity. At the same time, there is an urgent need to incorporate sustainable ingredients such as whey, a dairy by-product, into value-added formulations. Therefore, this study seeks to address these limitations by applying electrohydrodynamic atomization (EHDA) to encapsulate *L. reuteri* in chia–alginate hydrogels, aiming to preserve viability and functionality in a stable powdered beverage system.

Electrohydrodynamic atomization (EHDA), also known as electrospraying, has established itself as an emerging technology that allows the formation of monodisperse microcapsules at room temperature, making it a particularly suitable technique for thermosensitive compounds such as enzymes and probiotics [4,5,6]. EHDA is a non-thermal process that uses a high-voltage electric field to disperse a polymeric solution into fine droplets. These droplets are then solidified by cross-linking or dehydration, enabling the formation of uniform microparticles with tunable size and morphology. EHDA is especially advantageous in food applications for protecting labile bioactives under mild conditions. This technique has gained increasing attention due to its ability to generate particles with high homogeneity and dimensional control [7]. In this regard, EHDA is considered a promising technique for the encapsulation of various thermolabile compounds, such as enzymes and probiotic strains, since it operates without the use of heat and enables the formation of uniform microcapsules, thereby maintaining the viability of probiotics and improving their stability against gastrointestinal stress [8,9]. Studies have demonstrated its efficacy for strains such as *Lactobacillus plantarum*, *Bifidobacterium animalis*, *L. rhamnosus*, and *Lactobacillus fermentum,* helping to improve survival during gastrointestinal digestion, promoting adhesion to the intestinal mucosa, and allowing for a controlled release in the intestine [8,10,11]. Furthermore, the use of natural coating materials, such as sodium alginate and chia seed mucilage, contributes to improving the protective barrier and modulating the release kinetics. *Salvia hispanica* seed mucilage and sodium alginate in this study were carefully selected based their proven biocompatibility, film forming ability, and synergistic gelling properties. These polymers have demonstrated excellent performance in forming hydrogel networks that entrap probiotics and preserve their viability under gastrointestinal stress and processing conditions. Studies have reported that their combination improves encapsulation efficiency, modulates release kinetics, and enhances the mechanical stability of the capsules, making them suitable carriers for functional food applications [12].

In addition to the technological advantages of EHDA, it is crucial to consider the use of sustainable food matrices. In this context, the dairy industry is positioned as one of the main generators of by-products, with whey being one of the most abundant. According to Silva e Alves et al. (2018) [13], it is estimated that the global dairy industry produces approximately 200 million tons of whey per year, which has a high nutritional value containing lactose, calcium, phosphorus, folic acid, vitamins, and high-quality proteins [14], making it a functional matrix with great potential for the development of new fermented or functional beverages. In addition to the above, in recent years, various whey-enriched beverages have been developed, valued not only for their nutritional profile but also for their low cost and potential sustainability. Within this framework, the present study aimed to evaluate the encapsulation efficacy of *Lactobacillus reuteri DSM 17938* electrohydrodynamic spraying (EHDA) and dripping mode, with sodium alginate and chia seed mucilage as coating materials. The resulting encapsulates were added to a whey-based functional beverage flavored with pear and red berries, which was subsequently dehydrated by lyophilization for preservation and characterization.

Although prior research has demonstrated the potential of probiotic encapsulation through various techniques, the present study introduces a novel integration of electrosprayed *L. reuteri* into a real powdered beverage system based on dairy by-products, a combination not previously explored in this depth. Moreover, structural and functional characterization using advanced spectroscopic tools, such as Raman spectroscopy, remains scarce in this context. Therefore, this study fills a methodological and application gap by evaluating not only the encapsulation process but also the behavior of the encapsulated probiotics within a functional matrix. This dual focus on process optimization and matrix-level analysis distinguishes our approach from prior works and contributes new knowledge to both probiotic delivery and sustainable product development.

Unlike previous studies, this research presents an integrated approach that combines the encapsulation of *Lactobacillus reuteri* in chia–alginate hydrogels using electrohydrodynamic atomization (EHDA) with the development of freeze-dried functional powders based on whey and milk matrices. The innovation lies in the application of encapsulated probiotics to a complete food system, which was structurally characterized by Raman spectroscopy to assess the impact of matrix components and drying conditions. Furthermore, the use of whey as a sustainable ingredient not only adds value to dairy by-products but also enhances the functional properties of the formulation. This approach bridges the gap between encapsulation technology and real food applications, offering a promising strategy for the design of next-generation functional beverages.

## 2. Results and Discussion

### 2.1. Encapsulation Efficiency (EE)

As shown in Table 1, the results obtained showed no significant differences (*p* > 0.05) between the two microencapsulation methods (drip mode (DM) and electrohydrodynamic atomization (EHDA), with both achieving a viability of 9.9 log CFU/mL and an average EE of 99 ± 0.16%. The encapsulation efficiency (EE) values are expressed as mean ± standard deviation, based on three independent replicates (n = 3). No significant differences (*p* > 0.05) were found between the two encapsulation methods. These results suggest that both EHDA and DM provided a highly protective matrix for *Lactobacillus reuteri* during the encapsulation process. These values suggest that both encapsulation methods are effective in protecting and preserving *L. reuteri* cells during the microencapsulation process. This is possibly related to the characteristics of the polymers used (MC-AlgNa) and the type of structural network they form, which positively contributed to the microorganism’s survival. In addition, the nature of the coating may have facilitated some interactions, such as ionic or hydrogen bonds, which not only increased cell retention within the polymer matrix but also protected LR from adverse conditions during powder formulation. In this regard, Lee et al. (2003) [15] reported that the high effectiveness of these biopolymers is associated with an increased number of active calcium-binding sites in the polymer chains, resulting in a greater degree of crosslinking.

According to the above, several studies have reported that sodium alginate is a highly effective material for encapsulating probiotic bacteria, achieving high encapsulation efficiencies. In this sense, Perea-Flores et al. (2020) [12] reported that the combination of chia seed mucilage and sodium alginate as wall materials exhibited high entrapment efficiency (~94%) and provided a controlled release profile of the encapsulated compound. Although their study focused on rhodamine B, their findings highlight the potential of this biopolymer blend to protect sensitive compounds, suggesting it could be promising for applications involving probiotic encapsulation and protection under adverse conditions. Additionally, Popović et al. (2021) [16] reported a higher number of viable L. reuteri B2 cells encapsulated in sodium alginate combined with another biopolymer (sodium maleate), which improved their stability and viability.

### 2.2. Micromorphology Analysis

#### 2.2.1. Confocal Laser Scanning Microscopy (CLSM) Analysis

**Figure 1:**
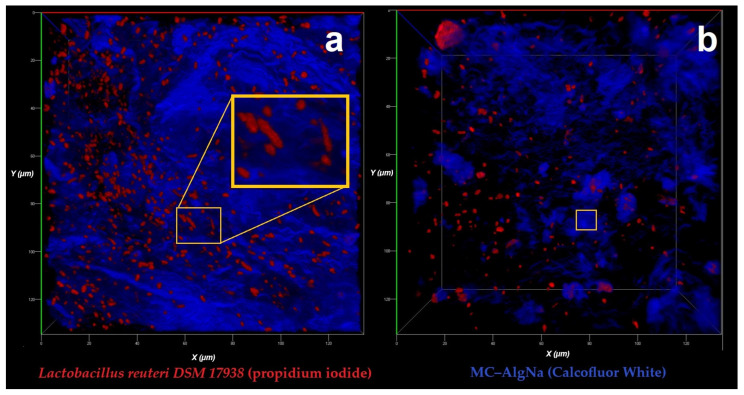
Confocal laser scanning microscopy (CLSM) images showing the distribution of *Lactobacillus reuteri DSM 17938* entrapped within the polysaccharide matrix composed of mucilage–alginate (MC–AlgNa). (**a**) Cells stained in red with propidium iodide; the orange box highlights a magnified region to emphasize bacteria embedded within the matrix. (**b**) Polymer matrix stained in blue with Calcofluor White.

The micrographs obtained (Figure 1a,b) show the interaction between *Lactobacillus reuteri DSM 17938* cells, the coating material (MC-AlgNa) and the fluorochromes (propidium iodide and calcofluor), where the uniform red fluorescence observed indicates that the bacteria remained embedded within the polymeric matrix of the biopolymers (MC-AlgNa), highlighting their typical bacillus morphology, with well-defined edges and a regular periphery. These morphological characteristics demonstrate that the encapsulation methods evaluated (EHDA and DM) did not cause deformations or damage to the bacterial cells. The smooth and uniform cell surface indicates an adequate interaction between the polymeric matrix and the cells, contributing to the protection of the bacteria against processing conditions.

In micrograph (a), a more uniform distribution of the bacteria in the matrix is observed. In contrast, in (b), the bacteria appear to be more dispersed, and some are grouped in specific regions. This difference is probably due to the rheological properties of the wall material during the formation of the capsules or to the specific operating conditions of each encapsulation method, as reported by Dragoni-Rosado, (2014) [17], who encapsulated *Lactobacillus casei ATCC 393* by extrusion and atomization methods, finding that extrusion tended to generate capsules with a more dispersed and clustered distribution of the bacteria, while atomization promoted a more homogeneous distribution, associating it with the forces applied during the process, which directly influenced the final distribution of the cells within the polymeric matrix. In addition, Huang et al. (2015) [18] demonstrated that similar biopolymeric matrices offer high biocompatibility and good cell retention. Furthermore, the absence of cell surface irregularities or deformations suggests that the processing conditions (EHDA, DM) did not negatively impact cell viability, which is crucial for prebiotic applications.

#### 2.2.2. Micromorphology Analysis by SEM

**Figure 2:**
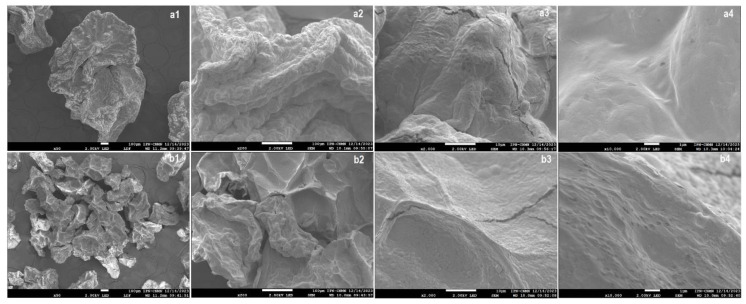
Scanning electron microscopy (SEM) images of microcapsules formulated with *Lactobacillus reuteri DSM 17938*. (**a1**–**a4**) Microcapsules obtained by the dripping method. (**b1**–**b4**) Microcapsules produced by electrohydrodynamic spraying (EHDA). Scale bars correspond to 200 μm in images (**a1**,**b1**), 100 μm in images (**a2**,**b2**), 50 μm in images (**a3**,**b3**), and 10 μm in images (**a4**,**b4**).

Morphological analysis of chia mucilage and sodium alginate microcapsules containing *Lactobacillus reuteri* (MC-AlgNa-LR) was performed by scanning electron microscopy (SEM) (Figure 2). The encapsulates prepared by electrohydrodynamic spraying (EHDA) had an average diameter of 1 ± 0.02 mm, while those obtained by the drip method showed a diameter of 2.5 ± 0.01 mm. Significant differences (*p* < 0.05) were observed between the two encapsulation methods (*p* < 0.05), which can be attributed to the conditions of microcapsule formation. After the lyophilization process, a reduction in particle size was observed, reaching values of 252 ± 0.02 µm for the microcapsules obtained by EHDA and 515 ± 0.003 µm for those prepared by dripping.

The encapsulates obtained by both methods (DM and EHDA) presented a spherical shape, with a rough surface and some wrinkles, characteristics typically associated with the partial collapse of the polymer gel network during dehydration by lyophilization [19]. Similarly, irregular structures were observed in both encapsulates, likely due to the formation of an agglomerate with variable textures, an effect attributed to moisture loss during the dehydration process. Figure 2a1–a4 (DM) display corrugated structures with fractures, similar to those observed in electrohydrodynamic spraying (Figure 2b1–b4). However, a significant difference in terms of uniformity and porosity was observed, as this parameter appeared to be affected in the encapsulates made by EHDA. This may be because the fractions of the hydrocolloids used as wall materials first gelled on the surface due to the interaction of calcium ions, slightly increasing the roughness. This result is consistent with data reported by Nasiri et al. (2021) [20], who encapsulated *Lactobacillus casei* in alginate microcapsules containing wild sage seed mucilage using the emulsion technique. Their results demonstrated spherical beads with a rough surface due to the incorporation of wild sage seed mucilage.

On the other hand, greater porosity was observed in the encapsulates prepared by EHDA (Figure 2b4), which was associated with the applied voltage, creating an electrostatic force that stretches the material and generates much finer, electrostatically charged droplets, thus promoting a much faster gelation compared to DM. Moreover, during lyophilization, these structures tend to retain and amplify the porosity channels initially formed. While in DM, the droplets are larger and more compact, with less interaction with the gelling solution, resulting in a denser network with fewer pores, as reported by Hernández San José, C. (2018) [21]. Humidity plays a critical role in the structural integrity and stability of microcapsules. Although the lyophilized powders obtained in this study exhibited low residual moisture, the porous structure observed especially in EHDA-derived microcapsules may increase their susceptibility to water uptake under high humidity conditions. Biopolymers such as alginate and chia mucilage are hygroscopic and tend to swell upon moisture absorption, which can lead to capsule deformation, reduced mechanical strength, and premature release or degradation of encapsulated probiotics. Similar phenomena have been reported in alginate-based hydrogel systems stored under uncontrolled humidity, where microcapsule stability deteriorates over time [22,23]. In agreement, Rajam et al. (2012) [24], demonstrated that probiotic-loaded microcapsules stored under humid conditions showed significant structural collapse, decreased cell retention, and early release of encapsulated *Lactobacillus plantarum* during storage. These findings underscore the importance of controlling environmental moisture to preserve probiotic functionality. Therefore, to maintain probiotic viability and microcapsule performance, it is essential to store these powders in low-humidity environments using moisture barrier packaging.

### 2.3. Physicochemical Properties of the Functional Beverages

**Figure 3:**
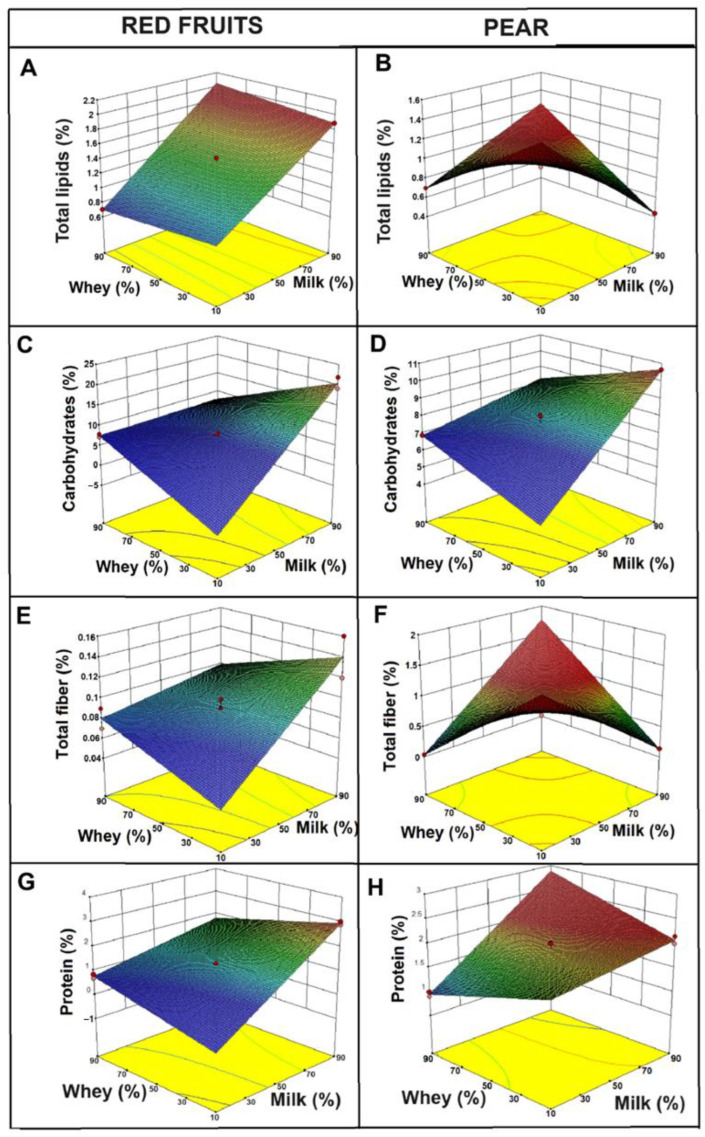
Response surface plots showing the effect of whey and milk concentrations on the physicochemical properties of beverages flavored with red fruits (**A**,**C**,**E**,**G**) and pear (**B**,**D**,**F**,**H**): total lipids (**A**,**B**), carbohydrates (**C**,**D**), total fiber (**E**,**F**), and protein content (**G**,**H**).

Figure 3 shows response surface graphs for different physicochemical properties of beverages made from whey and milk, flavored with red fruits (columns A, C, E, G) and pear (columns B, D, F, H). Each graph evaluates a specific property: total lipids, carbohydrates, total fiber, and protein, depending on the concentrations of whey and milk. The graphs were generated from a statistically validated 3^2^ factorial design using Design Expert *^®^* v10 software, with milk and whey concentrations as independent variables. Although the regression parameters are not displayed in the figure, the models showed strong predictive ability (R^2^ > 0.90), supporting the interpretation of the plotted surfaces. Regarding the total lipids present in the beverages (Figure 3A,B), it was observed that for red fruits (A), there was a linear increase with the proportion of milk, confirming that milk contributes the greatest amount of fat to the beverage. In contrast, the pear beverage (B) showed a peak in the intermediate combinations of milk and whey, suggesting that a possible synergistic effect between whey and milk components, or better homogenization, might have influenced the total lipid content. In addition, these differences are likely associated with the intrinsic composition of the fruits and their interaction with the liquid bases.

Similar studies have been reported by Chacón-Gurrola (2017) [25] in the physicochemical analysis of whey and milk, obtaining values of 0.3 g of fat content, while Shiby et al. (2013) [26] reported a value of 0.2 g in the development of energy drink mixes based on whey and Blue Grapes (*Vitis vinifera*) juice. On the other hand, Sepúlveda Valencia & Peña Alvarez, (2002) [27] reported a fat content of 2.64% in a beverage made from sweet whey (a fermented drink added with passion fruit), which is higher than that observed in the pear formulation, which showed a percentage of 1.02%. This difference is possibly associated with the type of milk used in both studies. In addition, these differences can be attributed to other factors such as the composition of the fruit, since red fruits (strawberry, raspberry and blueberry) have a very low fat content (<1%) [28], whereas pears, although also low in fat, contain pectins and soluble fibers that could interact with the lipids present in the dairy matrix, altering their distribution. Pectin is also widely used to stabilize acidified milk products, thanks to electrostatic interactions between its negatively charged chains and positively charged milk proteins, which reduce syneresis and help maintain a homogeneous texture [13]. However, this interaction is less significant in berry beverages, where pectins and other polysaccharides are present in lower concentrations. According to previous studies, the interaction between the dairy matrix and soluble fibers can promote the formation of more stable emulsions in formulations containing fruits, such as pears [29,30]. This phenomenon may explain the peak observed in the intermediate concentrations of milk and whey in pear beverages.

Similar behavior was observed regarding total carbohydrates, with distinct trends between the two formulations (red fruits and pear), depending on the proportions of milk and whey used. In Figure 3C, the carbohydrate content increased linearly with the increase in the proportion of milk, suggesting that the lactose present in milk is the primary source of carbohydrates in this formulation. In contrast, in Figure 3D, total carbohydrates are lower compared to drinks with red fruits, although they also increase with the proportion of milk. These results are possibly associated with the composition of the fruits. In this regard, red fruits have a significant carbohydrate content, primarily in the form of simple sugars such as glucose, fructose, and sucrose.

Furthermore, lactose, the main carbohydrate present in milk, although constant in concentration, can form complexes or interact with polysaccharides (such as pectins from pear), potentially limiting the availability of free carbohydrates in pear-flavored beverages, which could explain the lower apparent concentrations observed [31]. Moreover, Liang et al. (2020) [32] report that pectin binds to casein micelles in acidified milk systems, reducing syneresis and modifying the release and apparent concentration of carbohydrates in the matrix.

In pear-based beverages, pectin could form networks with proteins and other components of the dairy matrix, retaining part of the sugars and decreasing their measured concentration. In this sense, similar studies have shown that the addition of strawberry or berry pulps modifies the carbohydrate profile in dairy formulations [33]. Moreover, Silva et al. (2020) [13] found that beverages made with pectin-rich fruits, such as pears, may exhibit a reduction in available carbohydrates due to the interaction of the protein and fiber matrix. Additionally, Arcila & Mendoza (2006) [34] reported a content of 7.62% total carbohydrates in their drink based on whey and amaranth, attributed the higher carbohydrate content in the whey they used in its preparation.

Regarding fiber, a linear increase was observed in the red fruit drinks (Figure 3E) as the proportion of milk increased, which is associated with a greater retention of soluble fibers in the dairy matrix. On the other hand, in the pear drinks (Figure 3F), the highest amount of fiber was observed at intermediate concentrations of milk and whey; this effect may be attributed to the higher pectin content in pears and its ability to form networks with milk proteins [35].

In addition to the above, it was observed that the protein percentage in the red fruit drinks showed a linear trend, indicating that proteins were directly derived from the proportion of added milk. However, in pear drinks, a non-linear effect was observed due to the presence of pectins and other soluble polysaccharides that can form three-dimensional networks with proteins, improving their stability. In this sense, Laurent and Boulenguer (2003) [36] demonstrated that the addition of pectin-rich fruits to dairy products enhances protein–polymer interactions, thereby increasing the stability and viscosity of the beverages. On the other hand, Zamani et al. (2020) [30] found that interactions between pectins improve the stability of pear drinks.

### 2.4. Determination of Moisture and Color

In order to determine the moisture content of the powders used to prepare berry and pear-flavored beverages, a significant change was observed (*p* < 0.05) in both powders. For the berry powder, the higher the whey concentration (10/90 milk/whey) and the lower the milk concentration, the higher the moisture content, with an average value of 3.60 ± 0.20%. The opposite effect was observed at lower whey concentrations (90/10 milk/whey), with a percentage of 2.53 ± 0.31%. A similar trend was observed in the pear flavored beverages, where the highest moisture content was reported at higher whey concentrations (4.13 ± 0.12%), and the lowest moisture content corresponded to the formulation with the highest proportion of milk (3.98 ± 0.13%) (Table 2). This behavior can likely be attributed to the high water content of whey, which is approximately 93–94% [37].

Furthermore, the higher whey concentration likely favored an increase in free water and, consequently, moisture content in the powders. The opposite effect occurred with increasing milk concentration, as the higher fat content tended to limit water retention by displacing available water within the matrix. Likewise, differences in the solids content of the fruits used may have influenced the results; in this sense, red fruits provide a higher amount of total soluble solids, which may contribute to a reduction in moisture, while pears, with a lower solids content, may favor water retention [29].

With respect to the color of the powders, the formulations with red fruits presented statistically different lightness (L), red–green (a) and yellow–blue (b) values than those of the pear drink (*p* < 0.0500) (Table 2). In the 10/90 formulation (milk/whey), the powders with red fruits reached L = 44.06 ± 5.44, while the pear drink reached L = 76.16 ± 10.47, evidencing a considerably darker color in the former. Similarly, the a parameter was higher in red fruits (11.21 ± 7.27) compared to pear (1.92 ± 0.56), indicating a more intense red blue. For its part, the b value was lower in red fruits (1.43 ± 1.44) compared to the pear drink (14.87 ± 11.66), reflecting a lower tendency towards yellow or bluish tones. This behavior was maintained in the other proportions (50/50 and 90/10), where lower L and higher a values were recorded in drinks with red fruits (Table 2). This pattern resulted from the high concentration of anthocyanins, water-soluble pigments present in red fruits (such as strawberries, raspberries, and blueberries), which generate dark purple tones and produce a decrease in luminosity and an increase in absorbance [38]. Furthermore, the relationship between anthocyanin intensity and CIELAB values has been documented as inversely proportional to L and directly proportional to a [39]. On the other hand, pear-flavored powders exhibited high L and low a values, reflecting their light color and absence of intense pigments, such as anthocyanins [38]. This behavior is characteristic of fruits with low phenolic pigment content and has been reported in studies involving clear juices and flavored dairy products.

### 2.5. Structural and Molecular Characterization of Reconstituted Beverages by Raman Spectroscopy

**Figure 4:**
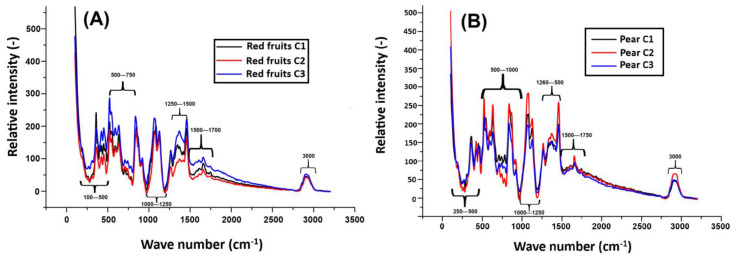
Raman spectra of functional beverages prepared with different concentrations of whey and milk. (**A**) Red fruit-flavored beverage: C1 (10% whey, 90% milk), C2 (50% whey, 50% milk), and C3 (90% whey, 10% milk). (**B**) Pear-flavored beverage: C1 (10% whey, 90% milk), C2 (50% whey, 50% milk), and C3 (90% whey, 10% milk). All formulations contain 20 g of stevia and 102 g of the corresponding fruit.

Regarding molecular characterization and interactions, Raman spectroscopy enabled the evaluation of the chemical composition of the reconstituted beverage from their respective powders. Figure 4A,B (pear and red fruit flavors) show the relative intensity of the Raman signal (Y axis) as a function of the wavenumber (X axis, in cm^−1^) for three samples labeled C1, C2, and C3. Several characteristic bands associated with the molecular vibrations of the compounds present in the samples were observed, allowing the identification of possible structural changes resulting from the addition of different ingredients and whey concentrations.

In the case of the pear-flavored beverage (Figure 4A), the peaks around 200–499 cm^−1^ are attributed to bond vibrations and heavy atom movements, such as those present in polymeric compounds or crystalline structures. These peaks are also associated with antioxidants and fruit-derived pectins. In contrast, in Figure 4B (red fruits), in this same region, fat-soluble fibers, antioxidants, and pectins present in the fruits (especially strawberries and blueberries) are highlighted, which contribute to the functional properties of the beverage and its structural stability. These results are consistent with those reported by Schulz and Baranska (2007) [40], who identified similar bands in fruits with high carbohydrate content, structural fibers, and pectins in fresh fruits.

On the other hand, in the pear drink, the vibrations observed in the 500–1000 cm^−1^ region are associated with the presence of iron, casein, and fat-soluble fibers, possibly originating from milk and whey. In this sense, for the red fruit powder, these peaks indicate the presence of iron, proteins, and casein, in addition to structural carbohydrates. This combination is typical of fruit-enriched dairy drinks. In the 1000–1250 cm^−1^ region in Figure 4A, pectins and key components of fruit cell walls, such as C-O-C groups and esters, are identified. These findings highlight the structural role of pectins in pear drinks. In contrast, in red fruit drinks, some compounds such as anthocyanins, sugars, and fats are more prominent in this spectral region. In this regard, Tadapaneni et al. (2012) [41] demonstrated that strawberry anthocyanins interact with dairy matrices, contributing to antioxidant functionality and influencing the stability of fruit-based dairy beverages.

In the 1250–1500 cm^−1^ region, the peaks observed in the pear-flavored beverage are associated with C–H bonds in methyl and methylene groups, as well as dairy proteins such as casein. Etzion et al. (2004) [42] reported that FTIR spectra exhibit characteristic amide I and II bands (1500–1700 cm^−1^) related to milk proteins, and that lipid-related vibrations (C–H stretching) appear in the 1400–1477 cm^−1^ region. Similarly, this region also indicates the presence of casein and milk fat. In the 1500–1750 cm^−1^ region, C = C and C = O stretching vibrations are associated with phenolic compounds, methylated pectins, and carboxylic acids. However, although these signals are present in the pear drink, a lower intensity is observed compared to the red fruit drink, since red fruits have a notably high content of phenolic compounds, especially anthocyanins and vitamin C, which are recognized for their antioxidant potential.

Finally, in the 2800–3000 cm^−1^ region, peaks related to O-H and C-H stretching vibrations were detected, which are characteristic of hydroxyl and methyl groups present in carbohydrates, lipids, and water. In the pear-flavored beverages, the higher intensity of these peaks was associated with the presence of total solids and a slight lipid contribution, although the fat content did not exceed 1%. This behavior is likely due to structural water retention and the possible formation of hydrogel networks resulting from interactions between proteins and pectins present in the beverage.

These structures are relevant for the development of gel-based systems capable of modulating the release of bioactive compounds and enhancing the physical stability of the product. These observations are consistent with the O-H and C-H group vibrations commonly found in carbohydrates and lipids, as reported by Schulz and Baranska (2007) [40]. In contrast, the red fruit-flavored beverages exhibited lower intensity peaks in this region, reflecting a compositional profile with a lower solids and fats content and a reduced ability to form hydrogel-like structures.

## 3. Conclusions

This study demonstrated that electrohydrodynamic spraying and dripping techniques are effective for encapsulating *Lactobacillus reuteri DSM 17938* using sodium alginate and chia seed mucilage as wall materials, achieving encapsulation efficiencies above 99%. The physicochemical characterization of functional powders made with different ratios of whey and milk revealed that matrix composition significantly influences moisture content, macronutrient distribution, and molecular interaction. Raman spectroscopy analysis revealed structural differences between pear- and berry-flavored beverages, highlighting the presence of pectin, casein, and specific phenolic compounds in the formulation. The highest intensity of the carbohydrate- and lipid-associated bands in the 3000 cm^−1^ region suggests the possible formation of hydrogel-like networks in the re-constituted beverages, particularly in the pear-flavored formulations. These hydrogels could promote water retention, structural stability, and controlled release of bioactive compounds.

These findings support the potential of combining dairy by-products with encapsulated fruit and probiotic ingredients to develop reconstituted functional beverages with enhanced structural, nutritional, and gelling properties. Future studies should investigate the rheological behavior, gel-forming capacity, and in vitro gastrointestinal release to validate the functionality and consumer acceptance of these formulations.

From a theoretical standpoint, this study contributes to the understanding of how hydrogel-based microencapsulation with chia mucilage and sodium alginate can enhance the stability and functional integration of probiotics into food matrices, particularly under freeze-drying conditions. Methodologically, it supports the use of electrohydrodinamic atomization (EHDA) as a non-thermal encapsulation technique suitable for sensitive probiotic strains, and it highlights the relevance of Raman spectroscopy and multiscale analysis (SEM, CLSM) for structural and functional evaluation of encapsulated systems.

## 4. Materials and Methods

### 4.1. Materials

Chia seeds (*Salvia hispanica*) and strawberries (*Fragaria ananassa*) were purchased from the local market in San Felipe del Progreso, State of Mexico. *Lactobacillus reuteri DSM 17938* (BioGaia, Stockholm, Sweden), De Man Rogosa and Sharpe (MRS) broth (Merck, 04053252452864, Rahway, NJ, USA), MRS agar (Merck, 110660), monobasic potassium phosphate (Sigma-Aldrich, 7778-77-0, St. Louis, MO, USA), sodium citrate (Merck, 6132-04-3), magnesium sulfate (Sigma-Aldrich, 7487-88-9), and glycerol (Merck, 104057) were used. Peptone water (Merck, 107228), sodium alginate (Deiman S.A. de C.V., 8060), CaCl_2_ (JT Baker, 1313-01, Phillipsburg, NJ, USA), propidium iodide (5 mg/mL; Sigma-Aldrich, 81845), and Calcofluor White stain (Sigma-Aldrich, 18909) were also utilized. Additionally, pasteurized whey and whole milk (approx. 3.5% fat content) were included as components of the beverage matrices. All reagents were of analytical grade and used as received, unless otherwise stated.

### 4.2. Methods

#### 4.2.1. Chia Seed Mucilage Extraction

Chia mucilage (CM) was extracted following the method previously described by Rentería-Ortega et al. (2021) [43]. Briefly, 15 g of *Salvia hispanica* seeds were mixed with 1000 mL of distilled water and stirred at 6000 rpm for 60 min at 40 °C. The mucilage was separated by pressing the hydrated seeds against a 40-mesh screen, and the resulting filtrate was precipitated using an ethanol–acetone solution (70:30, *v*/*v*). The extracted mucilage was dried at 40 °C for 4 h and it reached a final moisture content of 6.0%; then, it was milled and stored in sealed plastic containers for further use.

#### 4.2.2. Activation and Growth of *Lactobacillus reuteri DSM 17938*

The activation of the microorganism (*Lactobacillus reuteri DSM 17938*) was carried out in saline solution through serial dilutions according to that reported by Castro (2020) [44]. In the first tube, 100 μL of the commercial preparation *Lactobacillus reuteri DSM 17938* and 900 μL of saline solution were added and vortexed for 30 s. Then, in the second tube, 100 μL of the previous tube and 900 μL of saline solution were added, and it was vortexed again for 30 s. This process was repeated until eight serial dilutions were prepared, which were then incubated for 45 min at 25 °C. To determine the growth of *lactobacillus reuteri*, 1 mL of each dilution was transferred to Falcon tubes containing 9 mL of Man, Rogosa, and Sharpe (MRS) broth, resulting in a total of eight tubes. These tubes were incubated for 24 h at 37 °C, allowing for optimal development of the strain.

#### 4.2.3. Solution Preparation

For the preparation of the chia mucilage (CM), sodium alginate (AlgNa), and *Lactobacillus reuteri DSM 17938* (LR) solution, the methodology proposed by Sadeghi-Varkani et al. (2018) [45] was used with some modifications. The CM-AlgNa solution (0.5% *w*/*v*) was stirred at 6000 rpm (Thermo Scientific, Shanghai, China) for 15 min at room temperature (25 °C) until a homogeneous mixture was achieved. Then, *Lactobacillus reuteri* (6.98 × 10^10^ CFU/mL) was added by stirring until a uniform distribution was obtained.

The bacterial concentration was selected based on previous scientific recommendations and regulatory guidelines to ensure probiotic effectiveness. Burgain et al. (2011) [46] report that doses above 10^8^ CFU/mL are required to achieve gastrointestinal benefits. Additionally, Hill et al. (2014) [47] indicate that probiotic foods or supplements must contain at least 1 × 10^9^ viable cells per daily dose to be considered functional and properly labeled.

#### 4.2.4. Preparation of Encapsulates by Electrohydrodynamic Atomization (EHDA) and Drip Mode (DM)

The encapsulates were obtained using the electrohydrodynamic atomization equipment. The equipment was fed with the MC-AlgNa-LR solution, which was placed in a 10 mL syringe. The syringe was connected to the positive electrode of the current unit, with a voltage of 17 kV, a distance of 5.5 cm, and a flow rate of 2.0 mL/h. The collector was used to ground the system. To determine the preparation of the capsules made by DM, the same process conditions were used, except for the voltage. Finally, during the atomization and drip process, the encapsulates were collected from the CaCl_2_ solution and stored for later analysis [7].

#### 4.2.5. Encapsulation Efficiency (EE)

Regarding the determination of EE, the capsules were broken using the procedure described by Chávarri et al. (2010) [48], which involved combining 9 mL of sterile 100 mM sodium citrate solution, pH 7, with 1 g of capsules and vortexing until they were completely dissolved. Serial dilutions were then performed to obtain a countable number of cells. The dilutions were spread on Petri dishes containing MRS agar, and the dishes were incubated for 24 h at 37 °C for subsequent counting. The seedings were performed in triplicate. MRS agar (de Man, Rogosa, and Sharpe, Merk, Darmstadt, Germany; 110660) was used as the selective medium for the growth of *Lactobacillus reuteri*. This medium contains peptone, meat extract, yeast extract, dextrose, sodium acetate, triammonium citrate, polysorbate 80, dipotassium phosphate, magnesium sulfate, and manganese sulfate. The pH of the medium was adjusted to 6.5 ± 0.1 prior to autoclaving. After pouring the agar into Petri dishes, they were incubated in a humidity-controlled incubator at 37 °C for 24 h in aerobic conditions. Triplicate seedings were performed, and encapsulation efficiency was calculated as the ratio of viable cells recovered from broken capsules to the initial cell concentration. The colony-forming unit (CFU) was counted, and the encapsulation efficiency was determined according to the methodology reported by Mohammad et al. (2021) [49], which calculated the percentage of cells trapped in the capsule relative to the initial number of viable cells in the sample.

#### 4.2.6. Lyophilization of Encapsulates

The encapsulates were distributed in a Petri dish, frozen at −80 °C, and lyophilized using a 75,034 Bench Top Freeze Dryer (Labconco, Kansas City, MO, USA) for 24 h. The obtained powders were stored in amber bottles wrapped in aluminum foil until further analysis. Three individually prepared replicates were analyzed for each formulation.

#### 4.2.7. Microstructure Analysis

##### Confocal Laser Scanning Microscopy (CLSM) Analysis

The microstructure of chia seed and sodium alginate (MC-AlgNa) capsules loaded with *Lactobacillus reuteri DSM 17938* (LR) was evaluated by multiphoton confocal microscopy using an LSM 710 NLO (Carl Zeiss, Oberkochen, Germany). Staining of the probiotic and the coating material was performed before encapsulation.

The bacterial pellet (6.98 × 10^10^ CFU/mL) was resuspended in 1.5 mL of sterile distilled water, 500 µL of propidium iodide (5 mg/mL; Sigma-Aldrich, 81845) was added, and the pellet was sonicated in an ultrasonic bath (Branson-1510, Danbury, CT, USA) for 60 s at 40 kHz. It was then mixed with 16 mL of a solution containing 0.5 mg of chia mucilage and 0.5 mg of sodium alginate. Further, 2 mL of Calcofluor White (Sigma-Aldrich, 18909) was added to this mixture as a wall fluorochrome. The solution was transferred to 10 mL syringes (BD Plastipak, Dickson and Company, Franklin Lakes, NJ, USA) fitted with blunt-tip hypodermic needles (0.80 mm inner diameter) and subjected to electrohydrodynamic spraying (electrospraying), as described in Section 4.2.4.

Three-dimensional images of 100 sections were obtained using the LSM 710 NLO microscope, equipped with excitation lasers at 405 nm (for calcofluor) and 594 nm (for propidium iodide), and employing a Plan-Apochromat 63×/1.40 Oil DIC M27 objective. Image analysis was performed with NIS-Elements software (version 5.30.02; Nikon, Melville, NY, USA).

##### Scanning Electron Microscopy (SEM)

The capsules were characterized using micrographs obtained with a Field Emission Scanning Electron Microscope (JEOL, JSM-7800F, Akishima, Japan), with an acceleration voltage of 25 kV and a back-scatter electron detector (BSE). The capsules were lyophilized in a Freeze Dryer (ECO-FD10PT, LabTech, Sorisole, Bergamo, Sorisole, Italy) and sprinkled onto aluminum trays provided with conductive double-sided carbon tape and gold coating (Sputter coater, SPI-module, West Chester, PA, USA).

### 4.3. Beverage Preparation

The beverages were prepared following the methodology reported by Cuellas & Wagner et al. (2010) [50], with some modifications. Different concentrations of whey (10, 50, and 90%) and raw milk (10, 50, and 90%) were used, combined with fruit and a sugar substitute, stevia (20 g). The selected fruit proportions—98 g strawberry, 60 g raspberry, 45 g blueberry, and 203 g pear—were established through pilot-scale sensory screening to determine the most acceptable formulation when combined with the dairy matrix. These exact quantities were retained to ensure consistency and reflect the outcome of the optimization process. The mixtures were frozen at −80 °C and freeze-dried for 24 h (Labconco, model 75034, Kansas City, MO, USA). Afterwards, 10 g of *Lactobacillus reuteri DSM 17938* encapsulates were homogeneously mixed with 100 g of the lyophilized beverage powder, ensuring uniform distribution of the probiotic cells within the MC-AlgNa hydrogel matrix, as confirmed by CLSM imaging.

### 4.4. Physicochemical Characterization

#### 4.4.1. Protein

The crude protein content was determined using the Kjeldahl digestion method, with the standard nitrogen-to-protein conversion factor (N × 6.25). In detail, 15 g of each sample was weighed, and 0.4 g of copper sulfate pentahydrate, 6 g of potassium sulfate, and 20 mL of concentrated sulfuric acid were added as digestion reagents. The mixture was subjected to sequential digestion in a graphite digester (Hanon SH220, Hanon Instrument Co., Ltd., Jinan, China) at different temperatures and times: 120 °C for 30 min, 240 °C for 30 min, 360 °C for 1 h, and 420 °C for 1 h. The samples were then allowed to cool to room temperature. The determination of total nitrogen content was performed using an automatic Kjeldahl analyzer (ZDDN-II, Tuopu Instrument Co., Ltd., Hangzhou, China), according to the methodology reported by Lai Jing (2022) [51].

#### 4.4.2. Total Lipids

To determine total lipid content, 7 mL of each sample was added, followed by 90 mL of chloroform–methanol solution (2:1 *v*/*v*) at 45 °C for 2 h. A 30 mL NaCl solution (0.9%, *w*/*w*) was immediately added and mixed thoroughly. The chloroform was removed using a rotary evaporator at 40 °C (RE-52AA, Shanghai Yarong Biochemistry Instrument Factory, Shanghai, China). The remaining material was weighed using an electronic analytical balance (CP114, Ohaus International Trading Co., Ltd., Changzhou, China). It was expressed as a percentage of total lipid [51].

#### 4.4.3. Fiber

The fiber content was determined using the methodology reported by Guaman Rivera et al. (2023) [52], which involves sequential acid and alkaline digestion to remove proteins, lipids, and soluble carbohydrates. Briefly, 1 g of defatted sample was digested in 1.25% H_2_SO_4_ followed by 1.25% NaOH, with intermediate filtration and washing steps. The dried residue was weighed and then incinerated at 550 °C to obtain the ash content. The fiber percentage was calculated using Equation (1):

(1) $$Crude\;fiber\;\left(\%\right)=\frac{\left(W2-W1)-(W4-W3\right)}{W5}\times 100$$

where

W1 = Weight of filter paper (g);

W2 = Weight of filter paper + dried residue (g);

W3 = Weight of empty crucible (g);

W4 = Weight of crucible after incineration (g);

W5 = Weight of defatted sample (g).

#### 4.4.4. Ash Percentage

Ash content was determined using AOAC Method 923.03. First, the sample was weighed on an analytical balance (Precisa, model XT220A, Dietikon, Switzerland). After drying, the sample was incinerated at 550 °C in a muffle furnace (Selecta, model Select-furnace, Murten, Switzerland), and the incineration residue was determined by calculating the weight difference. The results are expressed as the percentage of ash calculated according to Equation (2).

(2) $$Ash\;(\%)=(\frac{W\;ash}{W\;sample})\times 100$$

where

W ash = Weight of ash after incineration (g);

W sample = Initial weight of the dried sample (g).

#### 4.4.5. Percentage of Total Carbohydrates

The total carbohydrate content was determined by difference, using the proximate analysis data. The percentage was calculated according to Equation (3):

Carbohydrates (%) = 100 − (Moisture (%) + Protein (%) + Lipids (%) + Ash (%) + Crude fiber (%))(3)

### 4.5. Color

For colorimetric analysis, 0.3 g of freeze-dried beverage powder was taken from the total formulation weight (110 g: 100 g beverage base + 10 g encapsulates). This sample size was selected according to the specifications of the colorimeter and allowed for consistent evaluation across all replicates. The reflection spectrum readings of the powders were performed as described by Rentería-Ortega et al. (2023) [53], using a Konica Minolta CR-400 colorimeter (Konica Minolta, NJ, USA), which had been previously calibrated with a standard reference plate (Y = 93.7, x = 0.3159, y = 0.3324). Measurements were taken at five different points on each sample, and the results represented the average of these readings. The coordinates obtained in the CIELab color space were reported, where L* indicates lightness (values between 0 and 100), a* represents the chromatic component from green (−) to red (+), and b* corresponds to the chromatic component from blue (−) to yellow (+). All measurements were taken on a standard white plate background to minimize interference. The results were analyzed statistically using one-way ANOVA, with a significance level of *p* < 0.05, using SigmaPlot 12.5 software. The reported values are the average of three independent measurements taken for each experimental condition.

### 4.6. Humidity

The moisture content was determined using an Ohaus MB120 moisture analyzer (Ohaus Corporation, Pine Brook, NJ, USA), according to the manufacturer’s specifications. For each measurement, 0.500 g of sample was placed directly onto the equipment’s weighing pan. Humidity was determined at a constant temperature of 105 °C for a 10 min analysis time, and the final value was obtained as a percentage of mass loss.

### 4.7. Raman Spectroscopy

For Raman analysis, 0.2 g of the powdered sample was used, extracted from the same 110 g total formulation. This amount complies with the standard sample volume required for instrument calibration and spectral stability. The molecular and structural changes in the powdered beverage added with whey-based LR encapsulants under different times, ingredients, and methods were described using Raman spectroscopy (LabRAM model HR800, Horiba Jobin Yvon, Kyoto, Japan) equipped with an Nd: YAG laser (785 nm) at room temperature (25 °C) and an integration time of 6 s. Three spectra were obtained for each sample and averaged using Origen Pro 8 software (v8.0724, OriginLab, Northampton, MA, USA). A baseline was then created and smoothed using the Savitzky–Golay method with a 2nd-order polynomial and 12 reference points in each spectrum, as reported by Rojas-Candelas et al. (2023) [54].

### 4.8. Experimental Design

A face-centered rotatable composite statistical design was used for each whey and milk concentration, with five replicates at the center point and duplicates at the other points. Data were processed using multiple regression analysis, and statistical significance (*p* ≤ 0.005) was found for the model and its variables. Design Expert V9 software (Stat-Ease, Inc., Minneapolis, MN, USA) was used to independently analyze the effects of the variables on the responses of protein, fat, moisture, fiber, and carbohydrates.

## Figures and Tables

**Table 1:** Encapsulation efficiency (EE) and viability of *Lactobacillus reuteri DSM 17938* using different encapsulation methods. Results are expressed as mean ± standard deviation (n = 3). Different letters in the same column indicate significant differences (*p* < 0.05).

Encapsulation Method	Viability (log CFU/mL)	EE (%)
Dripping mode (DM)	9.90 ± 0.05 ^a^	99.00 ± 0.16 ^a^
Electrohydrodynamic spraying (EHDA)	9.91 ± 0.04 ^a^	99.00 ± 0.01 ^a^

**Table 2:** Humidity and color parameters (L, a, b*) of functional beverage powders flavored with pear and red berries containing encapsulated *Lactobacillus reuteri DSM 17938*. Different letters within the same row indicate significant differences (*p* < 0.05).

Parameter	Concentration (Milk/Whey)	Pear Flavor	Red Fruits
*Moisture (%)*	10/90	4.13 ± 0.12 ^a^	3.6 ± 0.2 ^b^
Color (L*)		76.16 ± 10.47 ^a^	44.06 ± 5.44 ^b^
(a*)		1.92 ± 0.56 ^b^	11.21 ± 7.27 ^a^
(b*)		14.87 ± 11.66 ^a^	1.43 ± 1.44 ^b^
*Moisture (%)*	50/50	4.6 ± 0.13 ^a^	3.8 ± 0.6 ^b^
*C*olor (L*)		74.91 ± 5.70 ^a^	41.58 ± 5.34 ^b^
(a*)		7.20 ± 9.00 ^a^	7.2 ± 8.87 ^a^
(b*)		0.53 ± 0.79 ^a^	0.53 ± 0.79 ^a^
*Moisture (%)*	90/10	3.98 ± 0.13 ^a^	2.53 ± 0.31 ^b^
*C*olor (L*)		71.43 ± 7.20 ^a^	44.11 ± 4.07 ^b^
(a*)		5.90 ± 4.10 ^b^	18.54 ± 4.57 ^a^
(b*)		1.00 ± 0.60 ^a^	0.63 ± 1.56 ^b^

## Data Availability

The original contributions presented in the study are included in the article, further inquiries can be directed to the corresponding author.

## Abbreviations

The following abbreviations are used in this manuscript:

CFU	Colony Forming Units
CLSM	Confocal Laser Scanning Microscopy
CM	Chia Mucilage
DM	Drip Mode
EHDA	Electrohydrodynamic Atomization
FTIR	Fourier Transform Infrared Spectroscopy
LR	*Lactobacillus reuteri*
SEM	Scanning Electron Microscopy
